# Influenza influencing democracy

**DOI:** 10.3325/cmj.2011.52.91

**Published:** 2011-02

**Authors:** Nataša Škaričić

The vaccine against the pandemic or so-called swine flu caused scientific, political, and public health controversy all over the world. In Croatia, the whole case told us a lot about the state of the health care system, scientific community, and level of democracy.

When the new flu season started in December 2010, I found myself in the same uncomfortable position as at the peak of flu hysteria of 2009, when the World Health Organization (WHO) proclaimed the pandemic (1). As I then reported on the problematic contract between Novartis and the importer, the Institute of Immunology in Zagreb (2), and tried to stimulate a public debate about this contract, many people thought that I could give them an honest advice on whether to receive a flu shot or not. In 2009 and 2010, I received a number of calls and Facebook queries from worried people – mostly with some chronic health problem, who were confused by contradictory pieces of information that they could not put together to make a decision.

As I am not a physician, I thought – can I give such advice? Of course, I concluded at first that I could not. However, is the discussion on the contracts between the vaccine makers and European countries in which the makers decline their responsibility for the side-effects meant only for physicians and pharmacologists? To me, it should not be; moreover, the content of such unusual contracts is completely outside of their competence. This is, in the first place, a global precedent, which should be discussed from ethical, legal, human rights, and political aspects, and finally from the aspect of the relationship toward the consumer. In addition, as many governments, particularly the Croatian government, invested much effort to hide the contract from the public, it is a question of suspending democracy. Therefore, we have to seriously ask ourselves – “What is going on here?”

I was aware of these issues more than a year ago and tried to explain them during my public appearances, when my colleague editors from TV stations asked me to talk, always in the company of physicians. In one of these TV talk-shows, I openly asked the editor why only physicians were asked to participate in such debates, but I am not sure that my point was taken by anyone.

The phenomenon of pandemic flu vaccine is a topic for multidisciplinary discussion, as well as for the discussion on the ethics and transparency of pharmaceutical industry’s actions. The fact that only physicians were called upon to discuss this issue speaks about the conservatism and lack of knowledge about these topics in our society.

So I decided to tell this to all those seeking advice: personally, I would not, out of principle, buy, consume, and least of all get treated by a product from which its own manufacturer distances itself legally, the more so because there is no rational argument for such precedent. In addition, before making any decision, I would insist that the relevant political body, in this case the minister of health, provides the public with a serious and comprehensive answer why the government accepts such conditions for the purchase of a pharmaceutical product. If such an answer was lacking, I surely would not take the vaccine. I would finally say that this is my reasoning, of a person who does not have any serious health problem, and that I think that those who have it should make a decision together with their physicians and consider the risks and benefits of not receiving the vaccine.

I think that I, as a person involved in the public debate about this issue, provided the people with the data that are important for making their own decision.

But let me remind you how all this came about.

In the middle of 2009, the world media started to report about the appearance of a mutated flu virus in Mexico (3), paralleled by ad hoc reaction of physicians and official institutions about unusually high mortality rate and pandemic potential of the new virus. This caused global hysteria, so that there were soon no media that did not report this as breaking news topic.

Almost at the same time, the WHO started monitoring the development of the flu as a pandemic, estimating thus a higher risk for the global population, and reported on the development of the vaccine, which should have been ready before the flu season. Anti-pandemic plans were organized, and the governments were advised to accept them. In a very short time, the key part of the global plan became the generation of sufficient reserves of the vaccine, which was still in development and production.

As people have a generally positive opinion on the authority of the pharmaceutical industry and as public panic was created, they did not notice that the whole scenario was not much different from launching a new Hollywood blockbuster.

No, everybody was very serious. The regulatory bodies, such as the Federal Drug Administration in the USA and the European Medicines Agency, provided the vaccine manufacturers with urgent market approvals, the governments made urgent decision on reserving large quantities of the vaccine in the making, and the media continued to report on each and every death caused by the flu – the world united its efforts in preparing for a global tsunami.

Only those following the topic very carefully noticed that in each country there was a physician or two who publicly spoke about the irrationality of the situation, and then about possible dangers of the vaccine that was prepared so quickly.

In September 2009, I received a document with parts of the contract between the Institute of Immunology in Zagreb and Novartis. I was stunned: the clauses of the contract clearly showed that the Institute, as the importer of the vaccine for the Republic of Croatia “accepts that in the state of the urgent production, the company cannot guarantee the efficacy of the product and its side-effects” and “trusts the company’s respectability,” etc, etc.

The conspiracy elements of the story aside, I thought that the Croatian public needed to be urgently informed about such purchase of a product aimed at massive public use, just as I would do if I learned that a car manufacturer did not want to assume responsibility for the quality of the brakes in a shipment of cars delivered to Croatia.

The information I published received a huge response in the media, but the official bodies behaved as it was a breach of a top military secret: they completely ignored the content of the contract and persistently repeated that the vaccine was safe.

Five days after publishing the first report, I found a person at the Institute who was willing to make an official interview – Dr Srećko Sladoljev, member of the Supervisory Board of the Institute. While preparing for the interview, Dr Sladoljev clearly said that he did not want to talk about the safety of the vaccine, as he did not have direct experience with the particular problem. He was ready to talk about the business aspect of vaccine procurement, ie, the fact that the Institute ordered a vaccine shipment of HRK 330 million (€ 45 million) without securing further contracts with the countries it targeted as markets for the vaccine.

The interview was published (4) and the topic of the vaccine took about 5% of the space, but Dr Sladoljev was suspended from work the same day, with the explanation that he “revealed a business secret about the contract between Novartis and the Institute.”

It was evident that the state, as the major share-owner of the Institute, directed its discontent at its employee rather than at the news reporter, although the employee only confirmed the information that had already been published. In parallel, the Minister of Health and Social Care, Dr Darko Milinović, continued with the campaign to demonstrate the quality and safety of the vaccine. His campaign was supported by the Croatian reporters, who informed on each death caused by H1N1 virus. These statistics included children with leukemia (5), patients with grave chronic disease, and many other high-risk groups. At the peak of the crisis, when the Minister realized that people were not coming to get vaccinated, he and his family received the vaccine in front of reporters and cameras. It did not help.

Never, not with a single word, did he explain the reasons to accept the purchase agreement with Novartis, not even when the Polish Minister of Health, Ewa Kopacz, refused to sign such a contract for her country (6).

Nothing happened also when the official bodies in the European Commission ordered an investigation into the possible collaboration of the pharmaceutical industry and the WHO in creating the swine flu crisis, and established that there were “elements of conspiracy” (7).

Dr Sladoljev was later fired from the Supervisory Board of the Institute. The business damage because the vaccine was refused at the market in Croatia and Serbia (the Institute bought the vaccine for Serbia, without a prior contract) has never been established, although it was estimated at around HRK 140 million (€ 19.2 million). I will not comment here on the details of possible corrupt activities related to the vaccine procurement in Croatia.

Let me go back to the beginning: this year we have the same situation, except for the global hysteria, which rather calmed down. In the middle of December last year, the new vaccine campaign started, with coordinated attention of the media, which continued to report on death cases. Many reporters used social networks to invite persons who experienced H1N1 flu complications to talk about them, although these complications are not essentially different from those caused by any serious viral pneumonia. I am not sure that the same reporters would use the same energy for the prevention of cardiovascular diseases by publishing stories about how painful and horrifying it is to experience a myocardial infarction.

Such a campaign, which aims to cover up the already known information on the pharmaceutical industry’s and physicians’ lack of ethics in creating the pandemic hysteria in 2009 and create new panic from suffering this year, is so deeply unethical and unprofessional that we have to ask ourselves if the health care administration has any common sense, let alone political responsibility. My experience described at the beginning of this essay confirms that the campaign completely disoriented and scared people, who in the end were not sure of what they were afraid – the flu or the vaccine.

It is an even greater problem that Croatia has shown, again and again, that it is a society of dogmatic and anti-intellectual authorities capable of repressive and aggressive action if necessary. The work suspension of Dr Sladoljev is such an action.

This time, it was not about the protection of ideology, but the protection of global industry and local business at the time of a very serious public health crisis, when nobody from the public sphere succeeded in making the government and the medical profession to provide answers about their behavior. Croatia was one of the first countries where the contract with pharmaceutical industry was discovered by the public but the epilogue is that the topic has not been discussed at all.

A great theoretician of science, Paul Feyerabend, wrote in his essay How to Protect Society from Science (8) about the need for the formal separation of the state and science, in the same sense as in the case of religion. He suggested the formation of democratically elected advisory bodies of lay people, who would give their opinion on important projects in science. Feyerabend argued that science was not a closed book but an intellectual discipline that could be questioned and criticized by anyone interested, and that it only seems complicated because of a “systematic fogging campaign” lead by many scientists.

If we in Croatia cannot stand up to the state that fogs the answers to questions about health, how are we going to reach the level where each authority could and ought to be questioned? This may be the greatest challenge for the Croatian media. Example: in one of the debates on pandemic flu vaccine on the national TV, I was faced with four physicians and a reporter, who asked me if I was a supporter of conspiracy theories about pandemic flu virus. When I said that I did not support anything, especially not conspiracy theories, but that I only demanded that the Croatian public was explained the reasons why the contract in question had been made, the reporter made a disappointed pause and then went on to talk to other guests in the studio. This seriously disturbed me because I realized that some ridiculous concept, such as the thesis that former USA vice- president Donald Rumsfeld (9) and the military power was behind all this, would be received with greater attention than the obvious truth in front of our eyes – that the state accepted evasion of legal responsibility by a big corporation and protected safe profit instead of its citizens.

In addition to the media, the medical profession holds great responsibility because it readily pretended to be competent on the topic, when it had at least restricted competence.

The case of the pandemic flu vaccine is a sad and unfinished story about how the centers of power, irresponsible state, and the media blunt common sense. This is a story on how modern medicine is torn into two opposite directions: one to make people dependent on itself and its new products and the other to deter them by creating mistrust and fear. Both directions deserve our full attention.
